# Isolation and detection of *Mycobacterium avium *subsp. *paratuberculosis *(MAP) from cattle in Ireland using both traditional culture and molecular based methods

**DOI:** 10.1186/1757-4749-2-11

**Published:** 2010-09-27

**Authors:** Pierre E Douarre, William Cashman, Jim Buckley, Aidan Coffey, Jim M O'Mahony

**Affiliations:** 1 Cork Institute of Technology, Bishopstown, Cork, Ireland; 2 Veterinary Department, Cork County Council, County Hall, Cork, Ireland

## Abstract

**Background:**

*Mycobacterium avium *subsp. *paratuberculosis *(MAP) causes a chronic gastroenteritis affecting many species. Johne's disease is one of the most widespread and economically important disease of ruminants. Since 1992 and the opening of the European market, the exposure and the transmission of MAP in cattle herds considerably increased. Improvements in diagnostic strategies for Ireland and elsewhere are urgently required. In total, 290 cattle from seven Irish herds with either a history or a strong likelihood of paratuberculosis infection were selected by a veterinary team over 2 years. Faecal samples (290) were collected and screened for MAP by a conventional culture method and two PCR assays. In order to further evaluate the usefulness of molecular testing, a nested PCR was also assessed.

**Results:**

*M. paratuberculosis *was isolated and cultured from 23 faecal samples (7.9%) on solid medium. From a molecular perspective, 105 faecal samples (36%) were PCR positive for MAP specific DNA. A complete correlation (100%) was observed between the results of both molecular targets (IS900 and ISMAP02). Sensitivity was increased by ~10% with the inclusion of a nested PCR for ISMAP02 (29 further samples were positive). When culturing and PCR were retrospectively compared, every culture positive faecal sample also yielded a PCR positive result for both targets. Alternatively, however not every PCR positive sample (n = 105, 36%) produced a corresponding culture isolate. Interestingly though when analysed collectively at the herd level, the correlation between culture and PCR results was 100% (ie every herd which recorded at least 1 early PCR +ve result later yielded culture positive samples within that herd).

**Conclusion:**

PCR on bovine faecal samples is a fast reliable test and should be applied routinely when screening for MAP within herds suspected of paratuberculosis. Nested PCR increases the threshold limit of detection for MAP DNA by approximately 10% but proved to be problematic in this study. Although slow and impractical, culturing is still regarded as one of the most reliable methods for detecting MAP among infected cattle.

## Background

Johne's disease caused by *Mycobacterium avium *subsp. *paratuberculosis *(MAP), is one of the most widespread and economically important disease of ruminants. It is a chronic granulomatous enteritis affecting primarily ruminants and many other species [1], which is characterised by persistent diarrhoea, weight loss and a protein enteropathy, followed eventually by death [2]. Most cattle are infected early in life by the ingestion of faeces, milk or MAP contaminated water. The relatively long incubation period is characterized by the excretion of MAP in faeces for months and years before clinical symptoms develop [3]. The exposure to contaminated faeces constitutes one of the main risk factors for MAP transmission within the herd.

Johne's disease causes worldwide economic losses to farmers and dairy industries in terms of milk and meat production. It is considered a serious disease for dairy cattle as there is no effective treatment and it's control is difficult due to the long latent period. In dairy herds, losses are associated with reduced milk yield and weight gain, lower reproductive efficiency, premature culling and reduced values of culled cattle [4]. The effect of paratuberculosis on dairy operations in the USA was estimated at around $200 to $250 million a year [5].

Paratuberculosis has been a scheduled and notifiable disease in Ireland since 1955. Prior to the 1990 s, the low number of notified clinical cases (only 92 diagnosed between 1932 to 1992, mostly in imported animals) indicated that MAP wasn't widely established in the country. The importation of 85,000 cattle from continental Europe between 1992 and 2004 (Central Statistics Office, personal communication) as a consequence of the opening of the single European market in 1992 coincided with an increase in the prevalence of MAP infection in Ireland. Recent studies have shown that the risk of MAP exposure and transmission increases annually [6]. Between 1995 and 2002, 232 animals infected with MAP among 106 Irish herds were reported [7]. Also, in 2005, the seroprevalence of infected herds in Ireland was found to be 21.4% [8]. Currently, the prevalence of paratuberculosis among herds in Ireland is lower than that reported in many countries in Europe (Denmark 55%, France 68% and Netherlands 54%) [9] probably because of the late introduction and establishment of JD in Ireland. However it is likely that the prevalence in Ireland will continue to rise to match rates seen elsewhere, unless appropriate preventive and control measures are taken.

Similarities between Johne's disease in ruminants and Crohn's Disease (CD) in humans [10] as well as studies which identified MAP in CD's patient [11] have led to speculation that MAP may be a aetiological agent in Crohn's disease. Many reports showed the pathogenesis of CD is complex and multi-factorial with genetic and environmental contributions [12,13]. The causative link between MAP and CD is still controversial and the zoonotic potential of MAP remains a subject of debate [14]. The identification of viable MAP in pasteurized milk [15,16] and meat [17] should be regarded as a significant issue in terms of bio-security.

Detecting the presence of MAP is difficult because of the slow growth and the lack of sensitive tests to identify subclinically infected cattle. Specific and sensitive diagnostic tools as well as a better understanding of the pathogenesis of JD are needed to develop control programs to eradicate the disease.

This study was carried out on bovine faecal samples from 7 Irish herds which either had a history of or a likely exposure to MAP within 2 years of this study. The aim of this project was to evaluate a diagnostic strategy for MAP in targeted Irish herds. Specifically, we set out to compare a conventional culturing method with PCR, to evaluate the reliability of 2 different molecular targets and finally, to assess the improved sensitivity of a nested PCR assay.

## Methods

### Sample collection

**Figure 1:**
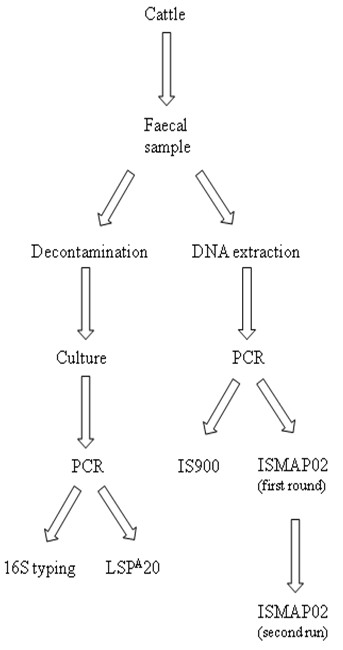
**Illustration of the sequential steps used as part of the sampling strategy for each animal**. Each Faecal sample was spilt upon collection in the lab and processed for culture (left arm) and molecular analysis (right arm).

290 individual bovine faecal samples were collected over 2 years (11/06 to 10/08) from 7 Irish herds which were strategically selected by a veterinary team. The location, the type of the herd, the breed and number of animals from each sampling period are summarized in Table 1. All the faecal samples were from individual animals, and were kept at 4°C up to 48 hours prior to processing for culture and DNA extraction. The strategy designed to process the samples is described in Figure 1.

**Table 1:** Information on the selected herds

Herd Id	Location	Type	No. animals	Breed	Sample date	n =
H1	Munster	Beef	140	^a^Mix	11/2006	4
H1	Munster	"	"	"	05/2007	22
H2	Munster	Dairy"	180"	Holstein Fr"	05/2007	44
H2	Munster				04/2008	73
H3	Munster	Dairy	630	Holstein Fr	07/2007	35
H4	Ulster	Beef	200	Mix*	07/2007	62
H5	Munster	Dairy	150	Holstein Fr	04/2008	17
H6	Munster	Dairy	150	Holstein Fr	04/2008	2
H7	Munster	Dairy	"	Holstein Fr	10/2008	31

Herd identification, location, type, breed and number of animals as well as the date and the number of samples tested.

^a ^mix of Charolais, Herford and Simmental breeds.

n = number of samples tested

### Sample preparation and culture

A modified centrifugation method was used to cultivate MAP [18]. Briefly, one gram of faeces was added to 20 ml of sterile distilled water, tubes were vortexed for 1 min and then allowed to stand undisturbed for 30 min. Five ml of the supernatant were added to 25 ml of 0.9% hexadecylpyridinium chloride (HPC) and allowed to stand undisturbed overnight at room temperature. Tubes were centrifuged at 1700 g (4300 rpm) for 20 min, the supernatant was decanted and the pellet was resuspended in 1 ml of 50 μg/ml amphotericin B. HEYM agar containing vancomycin, nalidixic acid and amphotericin B at 50 μg/ml were inoculated with 0.2 ml of the suspension and incubated in sealed 25 cm^3 ^tissue culture flasks (Sarstedt) at 37°C for 24 weeks.

### Confirmation of cultured mycobacteria

Slow growth rate and typical colony morphology was first observed before checking for acid fast bacteria and mycobactin dependency. Confirmation of the mycobacterial species was established by 16 S typing [19] and the presence of the MAP bovine specific large sequence polymorphism LSP^A^20 was detected by PCR [20]. In order to generate sufficient biomass, a single colony of each confirmed MAP isolate was subcultured into 7H9 broth supplemented with OADC and Mycobactin J and incubated at 37°C. Individual colonies from faster growing mycobacteria which were isolated during the sampling periods were also analysed by16 S typing as above.

As this study was designed to compare culturing with PCR, a representative sample from the same faecal sample was removed and processed as outlined below (see Figure 1):

### DNA extraction from faeces

One gram of faecal sample was added to 5 ml of 10 mM TE (pH 8.0), which was vortexed for 1 min and then allowed to stand undisturbed for 30 min. 750 μL of the supernatant was transferred to a 2 ml screw capped tube containing acid washed glass beads (Sigma, G1145) and 750 μl of fresh GITC lysis buffer (50 mM tris HCl, 10 mM EDTA, 2%Triton X-100, 4 M GITC, 0.3 M sodium Acetate) [21]. The samples were sheared in a Ribolyser (MagNA Lyser, Roche) for 45 sec at 6500 m.s^-1 ^and then boiled for 5 min. The tubes were then spun at 12000 rpm and the supernatant was transferred to a fresh tube. The disrupted cell extract was then mixed with an equal volume of phenol:chloroform:isoamyl alcohol 25.24.1. The samples were shaken manually for 5 min and centrifuged at 12000 rpm for 10 min. The aqueous layer was transferred to a fresh tube containing an equal volume of chloroform:isoamyl alcohol and shaken for 5 min. Following centrifugation at 12000 rpm for 10 min, the DNA was precipitated with 2 Vol of 100% ethanol and 0.5 Vol of ammonium acetate (7M) at -20°C overnight. After 2 washes with 70% ethanol, the DNA pellet was dried and resuspended with 50 μl 10 mM TE buffer.

### IS900 PCR

Samples were analysed by real time PCR targeting the IS900 element, using the method of O'Mahony (2002), [22] with modifications. Primer sequences for the amplification were 5'-GAAGGGTGTTCGGGGCCGTCGCTTAGG-3' and 5'-GGCGTTGAGGTCGATCGCCC ACGTGAC-3' (reverse primer) and generated a 400 bp product. The reaction mixture consisted of 2× LightCycler^® ^480 SYBR Green I Master (Roche) (containing the FastStart Taq DNA Polymerase, reaction buffer, dNTP mix, SYBR Green I dye and MgCl2), 0.5 μM of each primer and PCR grade water. Sample tubes contained 5 μl of faecal extract DNA. Controls consisted of reaction mixture alone (negative control) and a positive control containing 1 μl of genomic DNA from *M. avium subsp. paratuberculosis *(strain 19698). Samples were run according to the following conditions: 1 cycle at 95°C for 10 min and 35 cycles at 95°C for 10 s, 60°C for 10 s, and 72°C for 16 s. All PCR positive samples were then assessed by melting curve profile and conventional gel analysis.

### ISMAP02 PCR

Samples were run by using a real-time PCR targeting ISMAP02, a modified method of the PCR described by Stabel and Bannantine (2005), [23]. Specific primer sequences for the initial amplification were 5'-GCACGGTTTTTCGGATAACGAG-3' and 5'-TCAACTGCGTCACG GTGTCCTG-3' and generated a 278 bp product. The reaction mixture, samples and controls with the exception of the primers were prepared as described for the IS900 PCR. Samples were run according to the following conditions: 1 cycle at 95°C for 10 min and 35 cycles at 95°C for 10 s, 58°C for 10 s, and 72°C for 12 s. PCR results were confirmed as described above for the IS900 PCR.

### ISMAP02 Nested PCR

To evaluate the performance of a nested real-time PCR, the products resulting from the initial ISMAP02 PCR were re-tested by a second set of primers. The nested primers used for this second amplification reaction were 5'-GGATAACGAGACCGTGGATGC -3' and 5'-AACCGACGCCGCCAATACG-3' and yielded a 117 bp product. The reaction mixture, samples and controls were prepared as above. One μl of the amplicon from the first PCR was used as the template for the second amplification: 1 cycle at 95°C for 10 min and 30 cycles at 95°C for 10 s, 60°C for 10, and 72°C for 5 s. The size of the PCR amplicons was then confirmed as described above. To confirm (eliminate false positives) ISMAP02 nested PCR positive results, two alternative nested PCRs targeting IS900 (TJ1-4) [11] and F57 [24] were carried out on separate DNA extracts from samples which were ISMAP02 nested PCR positive.

## Results

### Isolation of viable mycobacteria by culturing

The culture results for all 290 faecal samples tested during this study are summarized in Table 2. MAP was cultured from 23 of the 290 samples tested (7.9%). Four herds (H1, H2, H3 and H4) were positive for MAP culture with at least 2 samples positive from each herd while the other 3 herds (H5,H6 & H7) were MAP culture negative. The percentage of MAP positive culture ranged from 3% (herd H4) to 75% (herd H1, 2006). The first 4 samples collected in 2006 from the herd H1 showed the highest percentage of culture positives and the presence of MAP in this herd was confirmed with the second batch, where typical MAP colonies were detected on solid media for 7 samples (30%). In relation to H2, 13.6% of samples tested in the first batch were MAP positive. The 73 samples tested 10 months later from the same herd were MAP culture negative but positive for other mycobacteria (18 isolates) as seen in Table 2. Using 16 S rRNA gene typing 17 samples were identified as *Mycobacterium non-chromogenicum *and 1 *Mycobacterium terrae*. Five cultures from herd H3 were confirmed to be MAP positive and only 2 samples (3.2%) in herd H4 showed MAP colonies indicating only a small number of viable MAP. Furthermore, MAP wasn't cultivated from samples recovered from herds H5, H6 and H7 but 4 other mycobacterial isolates were isolated in herd H5 (2 *M. non-chromogenicum*; 1 *M. hiberniae*) and H6 (1 *M. non-chromogenicum*).

**Table 2:** PCR and Culture Results

			No. of samples positive (percentage)				
**Herd Id**	**Sampling**	**No. of samples**	**Culture**		**PCR**		
			**MAP**	**Other**	**IS900**	**ISMAP02 (1)**	**ISMAP02 (2)**

H1	11/2006	4	3 (75)	0	4 (100)	4 (100)	4 (100)
	05/2007	22	7 (29.7)	0	22 (100)	22 (100)	22 (100)
H2	05/2007	44	6 (13.6)	0	44 (100)	44 (100)	44 (100)
	04/2008	73	0	18 (24.6)	0	0	6 (8.2)
H3	07/2007	35	5 (14.3)	0	14 (40)	14 (40)	20 (57.1)
H4	07/2007	62	2 (3.2)	0	21 (33.9)	21 (33.9)	34 (54.8)
H5	04/2008	17	0	3 (17.6)	0	0	1 (5.9)
H6	04/2008	2	0	0	0	0	0
H7	10/2008	31	0	1 (3.2)	0	0	3 (9.6)

**Total**		**290**	**23 (7.9)**	**22 (7.5)**	**105 (36.2)**	**105 (36.2)**	**134 (46.2)**

Data illustrating the results for each herd grouped according to PCR (IS900 & ISMAP02), nested PCR (ISMAP02), culture and non-MAP culture. In total seven herds were sampled over the 2 year period and the number of animals sampled ranged from 2 - 73. When examined collectively on a herd basis a 100% correlation was shown to exist between PCR and culture, ie the overall first round PCR results could be used to predict whether any animal in that herd would show up later as being culture positive (H1,2 ['07],3,4) or negative (H2['08], H5, H6, H7).

### IS900 PCR

The PCR results on the faecal extract DNA is summarized in Table 2. The IS900 target was detected in 105 samples representing 36% of the 290 samples tested. This target was detected in 4 herds H1, H2, H3 and H4, which correlates with the herd culture positivity rate. The percentage of positive samples varied from 100% (Herd H1 and H2) to 34% (herd H4). PCR-positive results were found in all 26 samples (100%) collected in herd H1 in 2006 and 2007. Similarly, all 44 samples tested (100%) from herd H2 were PCR positive in 2006. For the second batch of H2 samples collected 10 months later, MAP DNA was not detected, which correlates with the lack of culture positives from the same sampling period. In total, 14 and 21 DNA samples from H3 and H4 herds respectively were positive for MAP whereas all the samples from the 3 herds (H5, H6 and H7) were negative for the IS900 target.

### ISMAP02 PCR

Part of this study was to evaluate the performance and reliability of molecular testing. Consequently, in order to validate the results from above, a second MAP specific DNA target was chosen (ISMAP02). As with IS900, 105 samples were PCR positive as shown in Table 2 which indicates a complete correlation between the PCR results of both targets. In summary, of the 290 individual samples tested from 7 herds over 2 years, 105 were positive for ISMAP02 PCR. 100% of the samples in herd H1 (2006 and 2007) and H2 (2007) were ISMAP02 positive, whereas all the samples from the 3 herds (H5, H6 and H7) were negative. As seen previously for the IS900 PCR, 6, 14 and 21 DNA samples from H2 (2008), H3 and H4 herds were also positive for MAP respectively

### ISMAP02 Nested PCR

As part of this study wished to address the threshold limits of detection for MAP DNA, a nested PCR was evaluated using ISMAP02 as a target. In total 134 of 290 samples (46%) were positive after nested PCR. This number included 29 additional samples (10%) which were positive only after nested PCR. Of these (29), 6, 1 and 3 ISMAP02 positive samples were detected in the herds H2 (2008), H5 and H7 respectively. Interestingly, these 3 herds were all PCR-ve based on the first round analysis of both targets. The remaining nested PCR +ve samples, (6 and 13) were identified in herds H3 and H4 respectively

To confirm the validity of the nested PCR results, DNA from the 29 additional positive samples were re-tested with two independent nested PCR assays namely IS900 (with alternative primers, TJ1-4) and F57. Twenty one of these samples were confirmed as being true positives after the second run of the nested IS900 and F57 assays while 8 samples (all from the same herd H4) were negative, indicating a high likelihood of false positive nested ISMAP02 PCR results among this cohort only.

## Discussion

Due to the late introduction and establishment of JD in Ireland, there are relatively few publications on Paratuberculosis in this country. This study set out to evaluate a diagnostic strategy for MAP in selected Irish herds by comparing culture and molecular assays. This has relevance in terms of implementing future screening strategies for vets in Ireland and elsewhere. It is important to state that due to the logistical problems associated with obtaining samples, and the difficulty involved in enlisting willing farmers to participate, it was impossible to standardise the testing among herds in terms of obtaining equal samples from each herd. However, given this shortcoming, some important observations and conclusions can be made at the individual animal level and within each herd.

In general, we found a poor correlation between culture results from individual animals (7.9%) and the corresponding first round PCR results (36%). This may be due to the degree to which an animal was shedding the organism, the current disease status of the animal at the time of sampling, or the significant loss of viable cells during the harsh de-contamination step. Collectively however, it was interesting to note that all herds that tested positive by culture were also PCR positive (H1, H2, H3 and H4) whereas the other 3 herds were negative using both methods. This is significant in terms of how PCR may be used to manage and detect Johne's disease rapidly within a herd, i.e a quick and relatively cheap PCR test from a representative number of animals could provide an early indication of the disease status of the overall herd.

Two herds were sampled twice in this study (H1 and H2) and both produced interesting results. For example, in the herd H1, the percentage of MAP culture-positive results varied from 75% in 2006 to 30% in 2007 (although the sample size was not comparable). In terms of Herd H2, the difference between culture and PCR results in 2007 and the same herd one year later was striking (n = 44 and n = 0). This may be explained by the culling of infected animals and the possible introduction of "JD free" cattle in 2008, as seen previously by Richardson et al. (2009)[25]. It may also be explained by the selection of an alternative cohort of animals by the vets during the second visit. Due to the sensitivities involved in dealing with farmers involved in this voluntary study, limited information was made available to us regarding the degree to which the herd had been re-populated during the intervening year.

There is a significant lack of comparable studies in Ireland, and although the purpose of this project was not to draw conclusions regarding the prevalence of MAP in Ireland, it is interesting to note that our % recovery is almost double that seen by a similar study in 2002 (O'Doherty et al. in 2002[26]. Using a similar experimental strategy this group tested 221 animals from 16 herds suspected of paratuberculosis infection and found a prevalence rate of 4.1%.

As seen in other studies, the difference between the culture results in two dairy herds (H2, 13% and H3, 14%) and a beef herd (H4, 3%) was significant. The common practice in Ireland for dairy herd managers to feed pooled colostrums and milk to calves relatively increases the risk of transmission through contaminated milk which is a significant risk factor [27].

In terms of PCR and the reliability of molecular testing, a complete correlation was observed between the results of the IS900 and ISMAP02 targets. This was surprising but not unusual as observed in a similar study [23].

The nested ISMAP02 PCR detected 29 further positive samples increasing the sensitivity of the assay by 10%. However, among these additional samples, when re-examined, 8 from herd H4 were found negative by 2 other specific nested PCRs (IS900 and F57) based on independent triplicate results. The presence of these false positive test results may come from a single assay problem for H4, or may be representative of a larger reliability issue with nested PCR in this context. As well as the 8 putative false negatives, 10 of the remaining extra positives came from herds H2 (2008), H5 and H7 which were negative by both culture and first round PCR (Table 2). This may of course represent an excellent added sensitivity afforded by nested PCR for these 10 samples, or it could be indicative of potential problems with this extra test. We feel that the need for increased sensitivity must therefore be balanced with the risk of producing false positives among the results.

The presence of environmental mycobacteria (*Mycobacterium non-chromogenicum *and *Mycobacterium terrae*) within certain herds is another important finding although how it impacts on an animal's susceptibility to MAP infection is hard to assess (if at all). These mycobacteria belong to the M. terrae complex and were originally classified as non-pathogenic saprophytes, but their pathogenic status is changing. They have been isolated from soil and are usually present in clinical samples as an environmental contaminant. *Mycobacterium nonchromogenicum *has also been isolated in cattle infected by bovine tuberculosis [28]. Interestingly, the 18 samples from which *Mycobacterium non-chromogenicum *and *Mycobacterium terrae *have been isolated were PCR negative for MAP DNA which indicates that these two insertion sequence IS900 and ISMAP02 are not present in these mycobacteria.

Infected animals shed MAP in milk but also in the environment where MAP persists and survives in the soil, water and sediment [29]. To minimise exposure of the human population as described previously by Hermon Taylor (2010), there is a need to understand all the potential reservoirs and all the transmission routes for MAP. If this pathogen is proven to be zoonotic, the implication for the dairy industry worldwide would be enormous [30] especially in countries like Ireland where exported milk and dairy products are significant for the national economy. It follows therefore that all countries with a paratuberculosis related problem should be working towards a rapid, standardised and reliable indicator of infection. Based on our experience, PCR should play a significant role in this.

## Conclusion

In this study culturing of MAP was characterised by poor recovery (7.9%) but high specificity. In contrast, the PCR produced faster results with improved sensitivity (36%). The correlation between the culture and PCR result was poor at the individual animal level but in complete agreement at the herd level. Based on our experience, the choice of test employed by veterinarians should be guided by their objectives; a PCR test will produce a rapid result which may act as an "early warning" for the disease status of the herd especially if multiple animals are tested concurrently and periodically. As seen with other studies, IS900 and ISMAP02 are equally reliable as DNA targets for MAP, and the added sensitivity afforded by a nested PCR must be balanced with the potential for introducing false positive results. Despite the obvious advantages of introducing routine molecular testing for potentially infected animals, the culturing method (liquid or solid based) still remains the gold standard despite its logistical and practical limitations.

## Competing interests

The authors declare that they have no competing interests.

## Authors' contributions

PED carried out the culturing, molecular analysis and drafting of the manuscript. WC and JB were the veterinarians responsible for co-ordinating the sampling, collection and transport of the faecal samples. AC and JO'M are the principal investigators and grant awardees who designed and managed the study, as well as editing the manuscript.

All authors read and approved the final manuscript.
